# Supplementary material for: Naturally acquired blocking human monoclonal antibodies to Plasmodiumvivax reticulocyte binding protein 2b
Source: Nat Commun. 2021 Mar 9;12:1538. doi: 10.1038/s41467-021-21811-2 (PMC7943553; doi:10.1038/s41467-021-21811-2)
Supplement: Supplementary file 1 — Supplementary Information [file 41467_2021_21811_MOESM1_ESM.pdf]

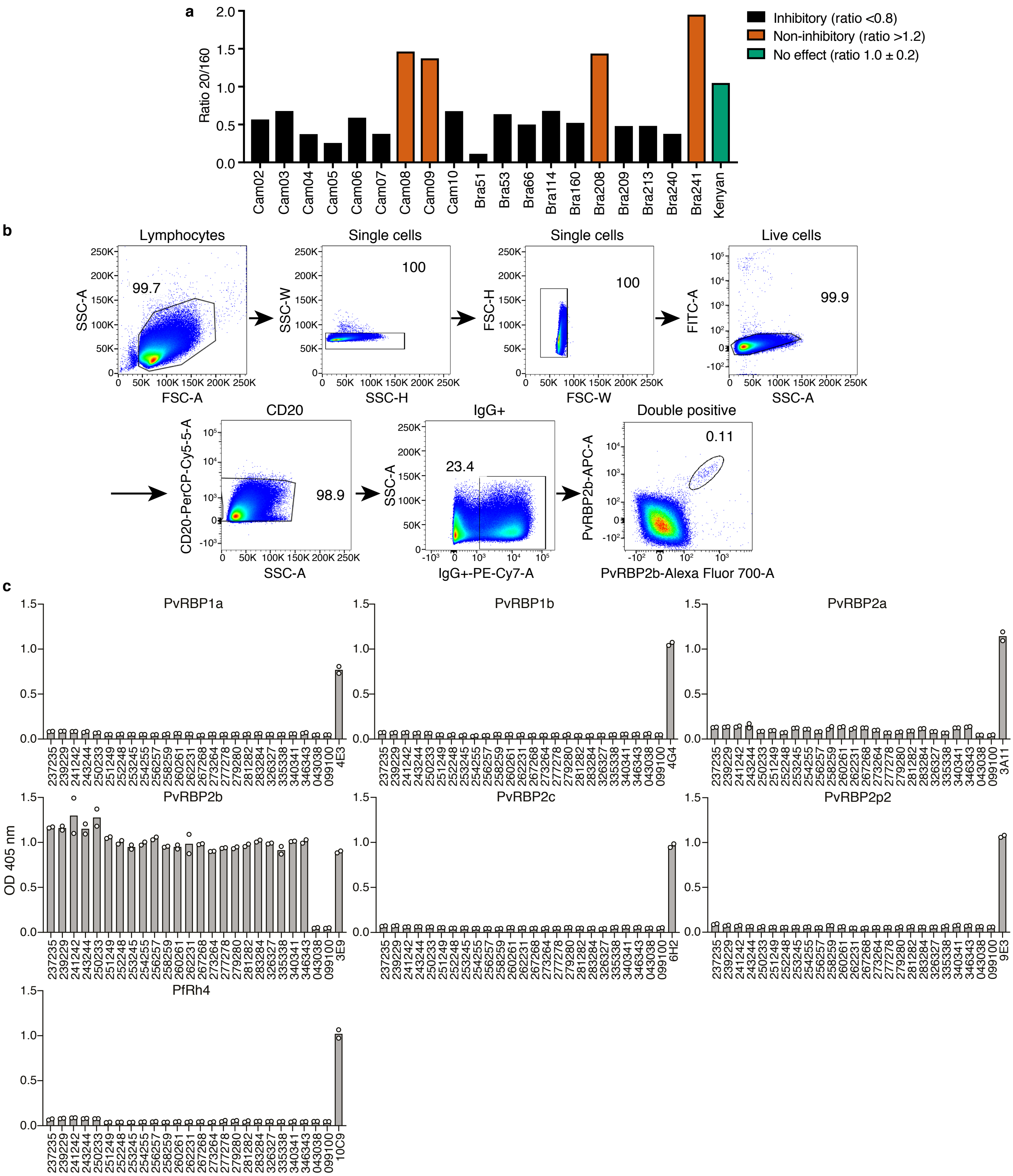

Supplementary Figure S1. Functional blocking antibodies against PvRBP2b in serum and antibody specificity of PvRBP2b human mAbs using ELISA. (a) PvRBP2b$_{161-1454}$ binding to reticulocytes in the presence of serum from Cambodian (Cam) and Brazilian (Bra) individuals and one Kenyan individual analyzed by flow cytometry. Binding results were expressed as a ratio between PvRBP2b$_{161-1454}$ binding at a 20-fold dilution of sera over a 160-fold dilution of sera, to account for non-specific increases in PvRBP2b$_{161-1454}$ binding in the presence of sera from individuals exposed to *P. vivax*. Black, ratio <0.8 and inhibitory; Orange, ratio >1.2 and non-inhibitory; Green, ratio 1.0 ± 0.2 and no effect. **(b)** Gating strategy for sorting PvRBP2b specific memory B cells. **(c)** Antibody specificity of PvRBP2b human mAbs to PvRBP family members and PfRh4 using ELISA. Bar graphs represent the mean of duplicate measures represented as circles. Mouse mAbs 4E3, 4G4, 3A11, 3E9, 6H2, 9E3 and 10C9 were used to detect the coating of PvRBP1a, PvRBP1b, PvRBP2a, PvRBP2b, PvRBP2c, PvRBP2p2 and PfRh4 on plates, respectively. Graphs are a representative of two independent experiments. Source data are provided as a Source Data file.

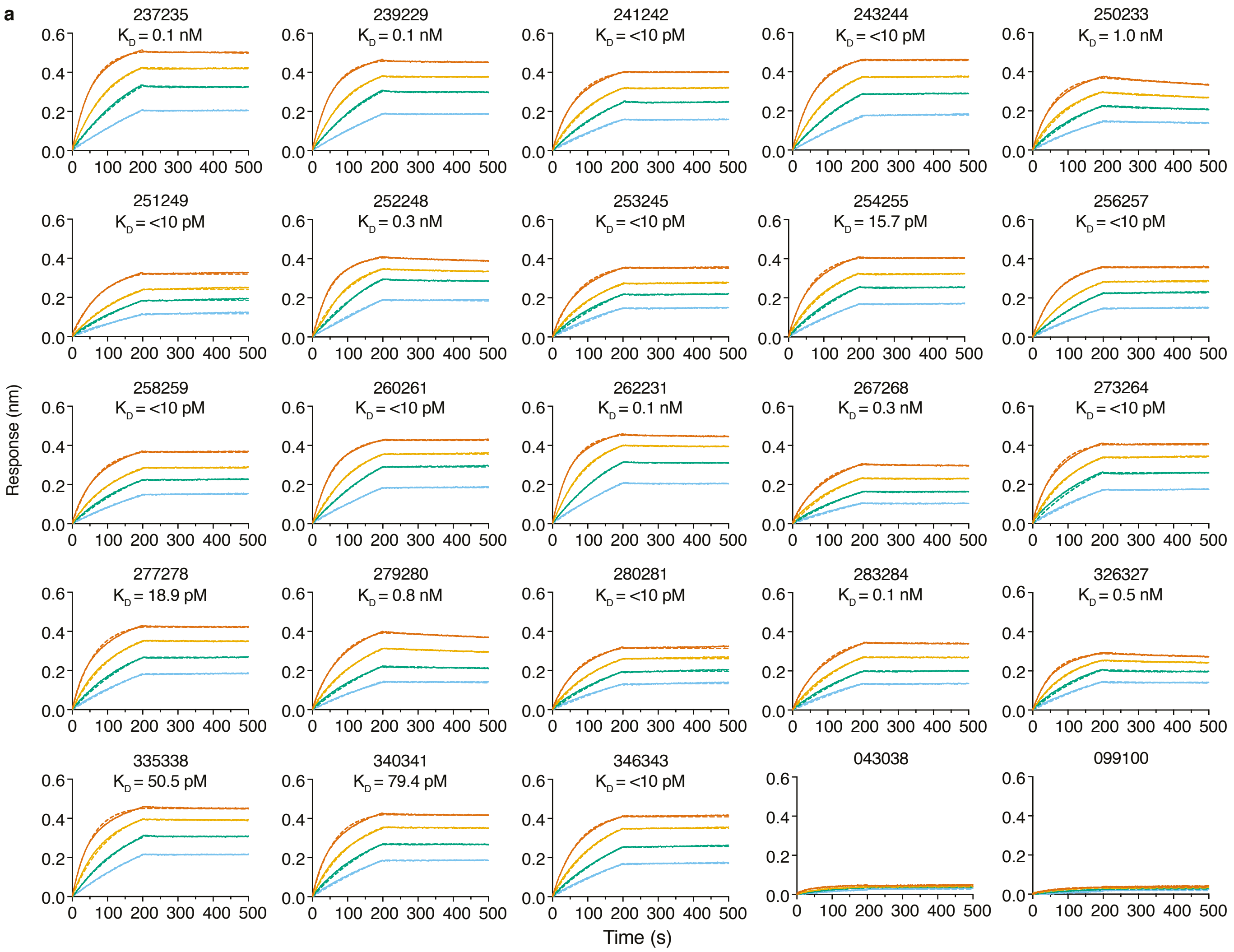

b

mAb	k_a ($M^{-1}s^{-1}$)	k_d (s^{-1})	K_D (M)
237235	3.70E+05	3.45E-05	8.83E-11
239229	3.87E+05	4.60E-05	1.12E-10
241242	3.21E+05	2.13E-05	6.10E-11
243244	3.37E+05	1.15E-05	3.66E-11
250233	3.53E+05	3.50E-04	9.88E-10
251249	2.71E+05	<1.00E-06	<1.00E-11
252248	3.42E+05	1.93E-04	5.86E-10
253245	2.73E+05	8.17E-06	3.07E-11
254255	3.01E+05	2.52E-05	7.42E-11
256257	2.84E+05	<1.00E-06	<1.00E-11
258259	2.88E+05	7.57E-06	2.69E-11
260261	3.29E+05	6.23E-06	2.05E-11
262231	3.98E+05	6.22E-05	1.40E-10
267268	2.40E+05	5.71E-05	2.13E-10
273264	3.27E+05	3.02E-05	8.30E-11
277278	3.76E+05	3.08E-05	7.96E-11
279280	2.60E+05	1.94E-04	7.27E-10
281282	2.63E+05	6.03E-06	2.73E-11
283284	2.26E+05	6.00E-05	2.46E-10
326327	3.78E+05	2.46E-04	6.42E-10
335338	4.32E+05	3.08E-05	7.82E-11
340341	3.37E+05	3.85E-05	1.07E-10
346343	2.82E+05	<1.00E-06	<1.00E-11

Supplementary Figure S2. PvRBP2b human mAb binding kinetics by bio-layer interferometry. (a) Representative sensorgrams (solid lines) and curve fitting analysis using a 1:1 model (dashed lines) for human mAbs binding to PvRBP2b$_{161–1454}$. A two-fold concentration gradient of PvRBP2b$_{161–1454}$ from 6 - 50 nM is shown by the different colored lines (50 nM, red; 25 nM, orange; 12 nM, green; 6 nM, blue). The calculated K_D is shown for each sensorgram. 043038 and 099100 are isotype controls. **(b)** Table of association rate constants (k_a), dissociation rate constants (k_d) and equilibrium dissociation rate constants (K_D) for PvRBP2b human mAbs. Table values are the mean of four independent experiments. Source data are provided as a Source Data file.

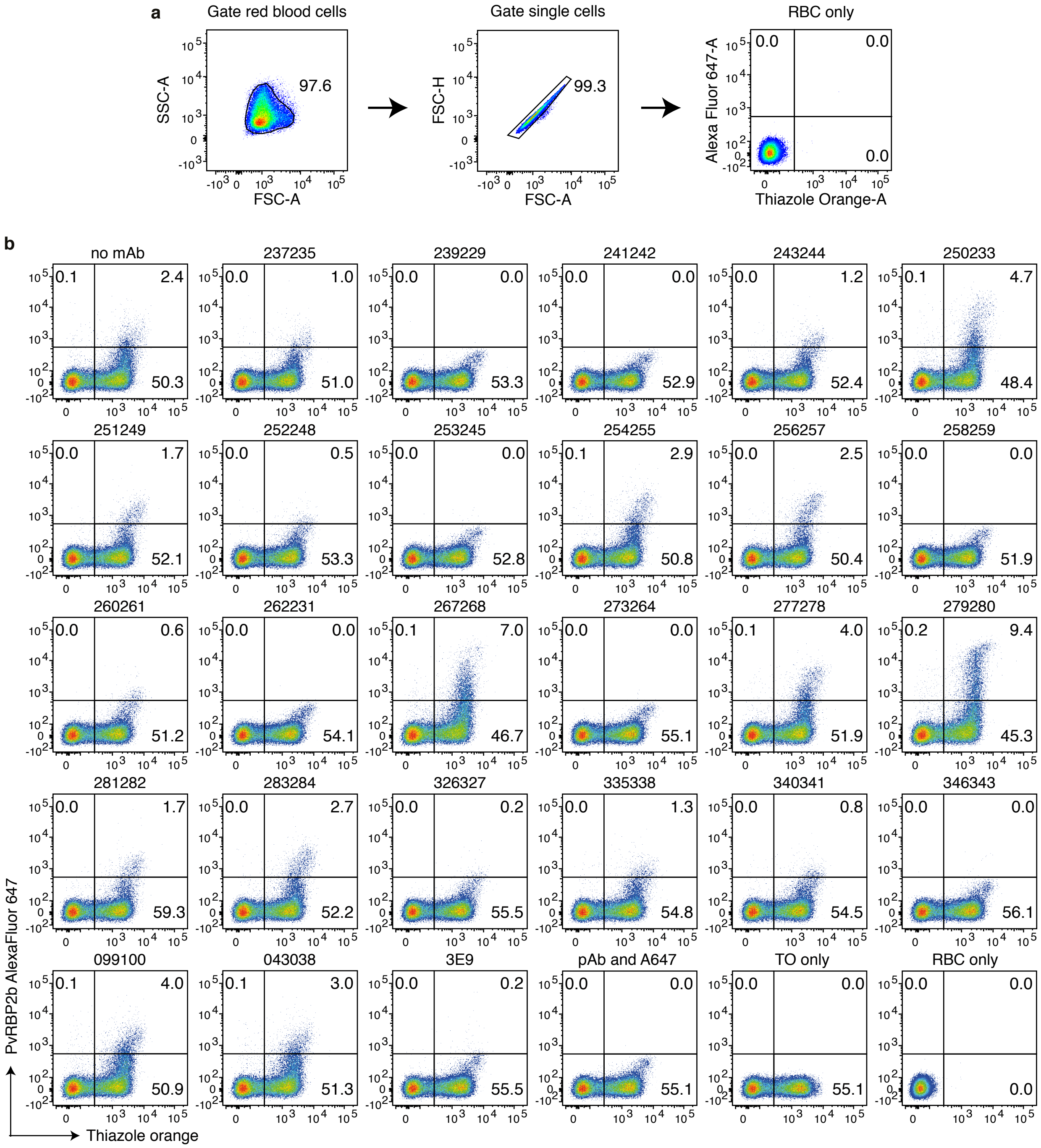

Supplementary Figure S3. PvRBP2b human mAbs block PvRBP2b binding to reticulocytes. (a) Gating strategy for red blood cells. Red blood cells were gated using forward (FSC-A) and side scatter (SSC-A). Single cells were gated using forward scatter area (A) and height (H). Thiazole orange nucleic acid dye (TO) was used to stain the reticulocyte population on the x-axis. PvRBP2b$_{161–1454}$ binding was detected with PvRBP2b polyclonal antibodies (pAb) and Alexa 647 secondary antibody on the y-axis. **(b)** Representative dot plots showing the binding of PvRBP2b$_{161–1454}$ to reticulocytes in the presence of PvRBP2b human mAbs. 099100 and 043038 were isotype controls and 3E9 was an inhibitory mouse mAb control. The reticulocyte population was gated on the red blood cell (RBC) population and the PvRBP2b$_{161–1454}$ positive population was gated on the detecting antibodies (pAb and A647) control that showed a background signal.

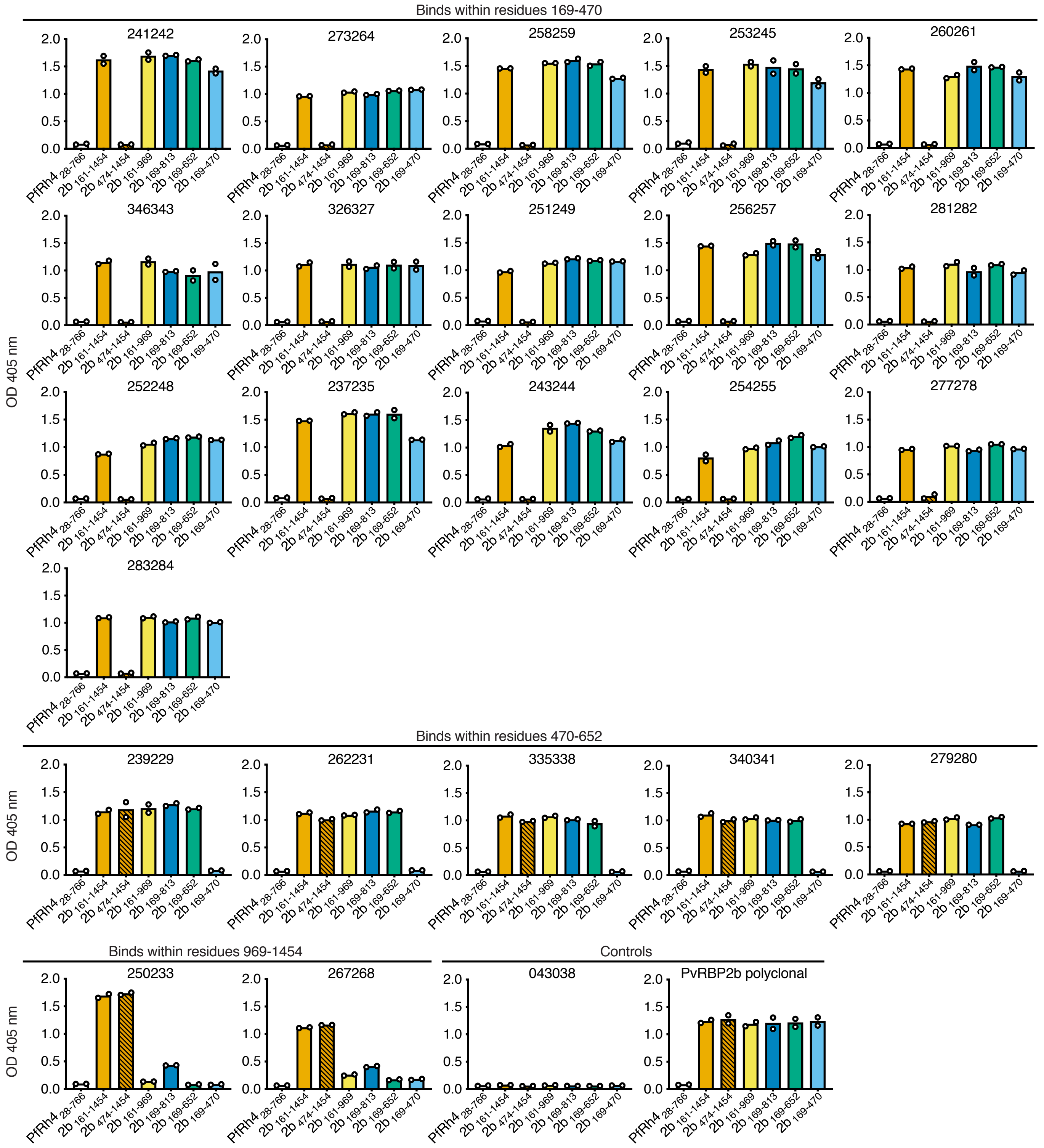

Supplementary Figure S4. Domain mapping of PvRBP2b human mAb epitopes. Binding of PvRBP2b human mAbs to PvRBP2b recombinant fragments and PfRh4$_{28\text{-}766}$ was detected by ELISA. PfRh4$_{28\text{-}766}$ was used as a control for non-specific binding of PvRBP2b human mAbs. PvRBP2b polyclonal antibody was used to detect coating of PvRBP2b fragments and 043038 was used as a negative control. Bar graphs represent the mean of duplicate measures represented as circles. Graphs are a representative of two independent experiments. Source data are provided as a Source Data file.

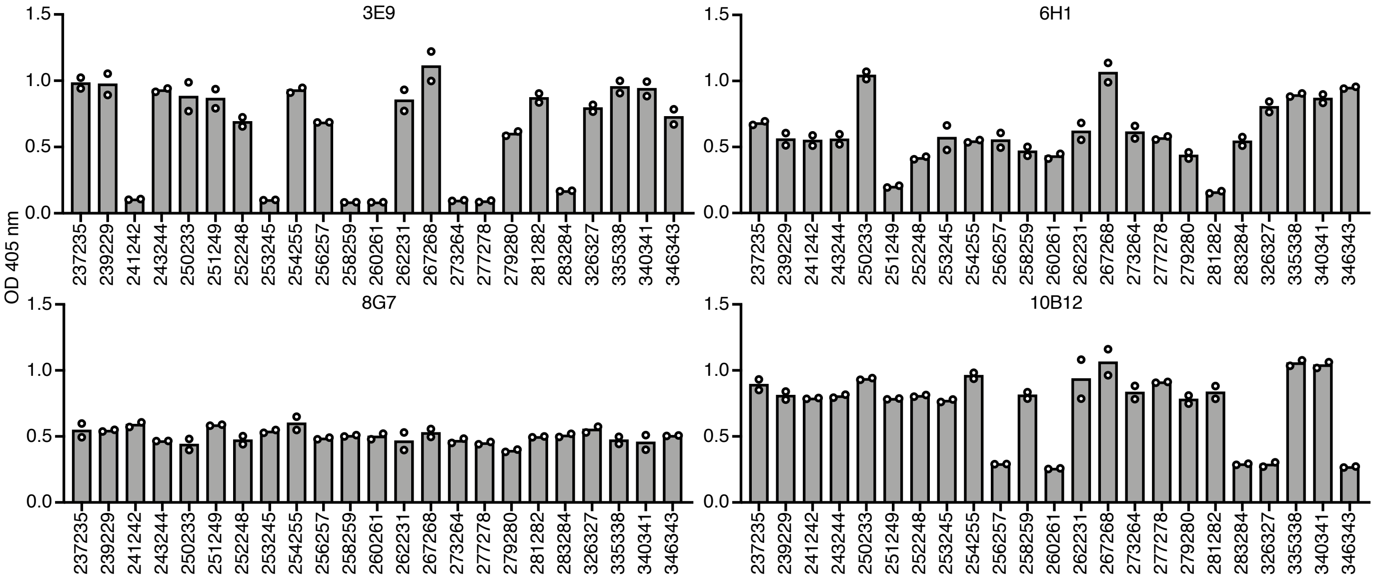

Supplementary Figure S5. Competition between PvRBP2b human mAbs with PvRBP2b mouse mAbs. Competition ELISA using immobilized PvRBP2b mouse mAbs incubated with a mixture of PvRBP2b human mAbs with PvRBP2b$_{161-1454}$ at a 20:1 molar ratio. Bar graphs represent the mean of duplicate measures represented as circles. Graphs are a representative of two independent experiments. Source data are provided as a Source Data file.

RBP2b peptide map

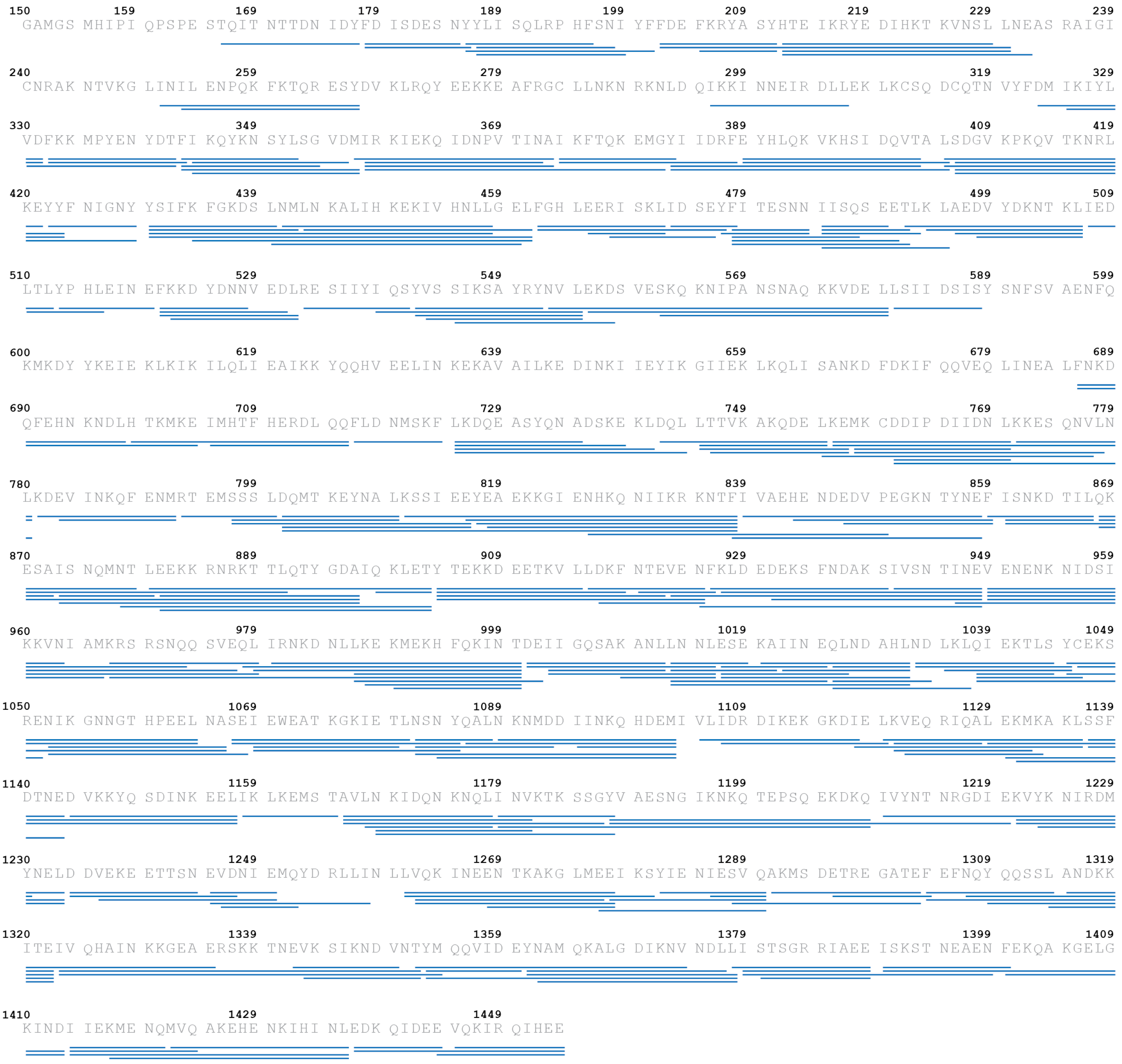

Supplementary Figure S6. HDX peptide map for PvRBP2b$_{161\text{-}1454}$. PvRBP2b$_{161\text{–}1454}$ peptide map showing the peptides used for HDX-MS analysis.

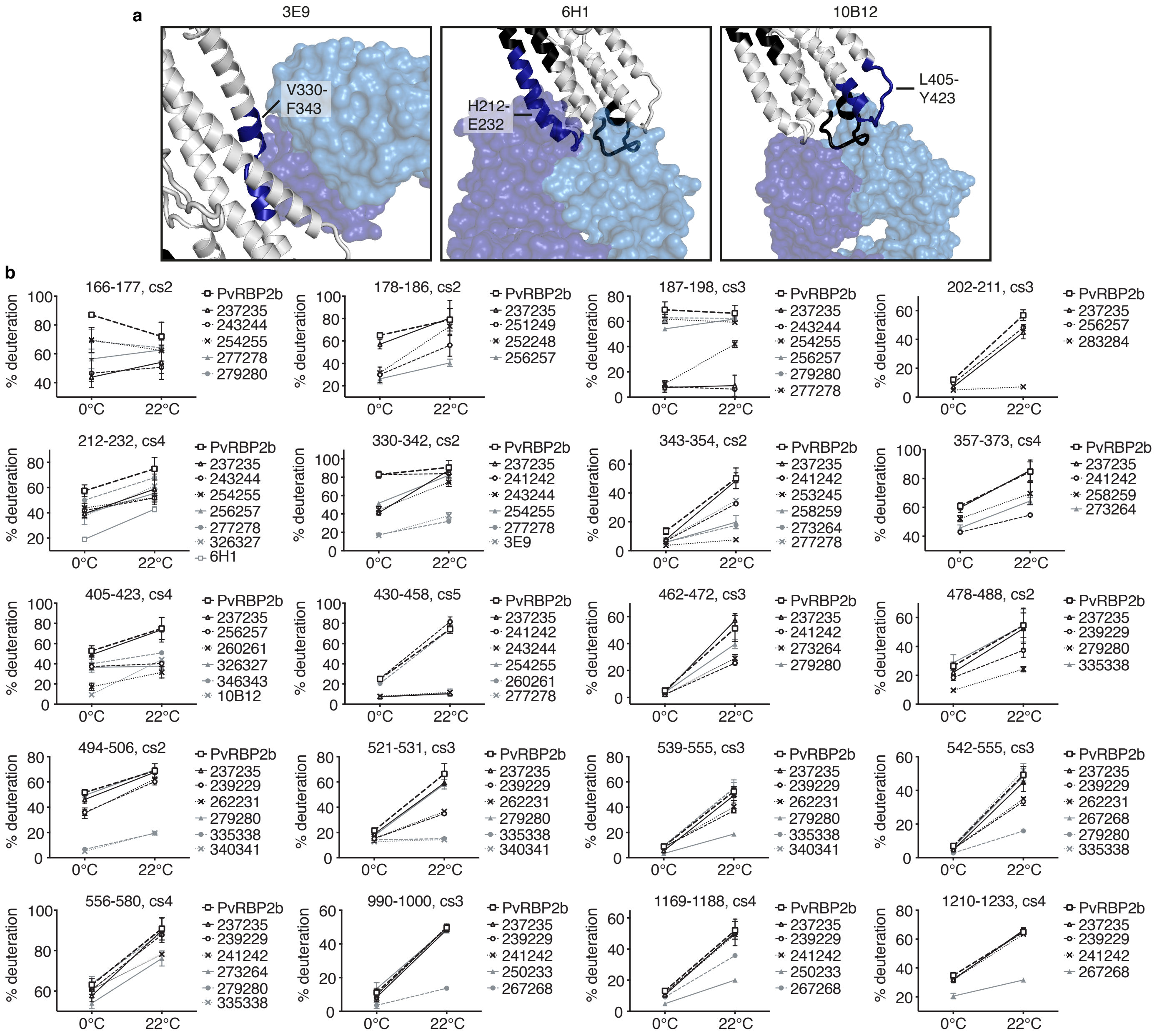

Supplementary Figure S7. Comparison between PvRPB2b epitopes identified by HDX-MS and X-ray crystallography for mouse mAbs and HDX-MS uptake plots for a selection of PvRBP2b peptides. (a) Combination of HDX-MS data with crystal structures of PvRBP2b mouse Fab fragments bound to PvRBP2b. Fabs shown in transparent surface representation. Regions that show protection by HDX-MS for each mAb are colored in blue and the residue range is labelled. Black indicates the regions where no peptides were detected by mass spectrometry. **(b)** Uptake plots showing deuterium incorporation levels for a selection of PvRBP2b peptides. For each peptide, the level of deuteration, expressed as percentage compared to a highly deuterated sample, is shown for samples incubated 5 min in deuterated buffer at 0°C and 22°C. One peptide representative of each of the PvRBP2b region showing protection in Fig. 4A is shown. Deuteration level of PvRBP2b alone is shown for every peptide. A selection of mAb showing either no change or a significant difference in deuteration level compared to PvRBP2b alone is shown for every peptide. n=3 independent experiments. Data are presented as mean ± SD. Abbreviation: cs: charge state of the analyzed peptide**.** Source data are provided as a Source Data file.

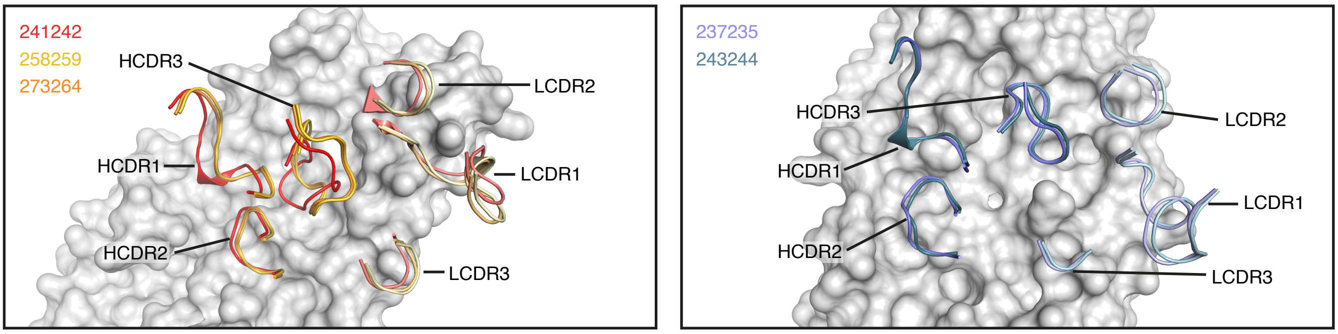

Supplementary Figure S8. Superimposed CDR loops of antibodies with overlapping binding sites. (Left panel) Superimposed CDR loop structures (HCDR; heavy chain CDR loops, LCDR; light chain CDR loops) of 241242 (HCDR: red, LCDR: pink), 258259 (HCDR: yellow, LCDR: light yellow) and 273264 (HDCR: orange, LCDR: light orange) (same clonal group as 258259) binding to PvRBP2b. (Right panel) Superimposed CDR loops of 237235 (HCDR: lavendar blue, LCDR: light lavendar blue) and 243244 (HCDR: slate, LCDR: light blue) (both from the same clonal group) binding to PvRBP2b.

a

150 ... 200 ... R217 ... R242 ... 250
GAMGSMHIPIQPSPESTQITNTTDNIDYFDISDESNYYLISQLRPHFSNIYFFDEFKRYASYHTEIKRYEDIHKTKVNSLLNEASRAIGICNRAKNTVKGLINILENPQKF

K288 ... 300 ... K309 ... 350 ... K363 D366
KTQRESYDVKLRQYEEKKEAFRGCLLNKNRKNLDQIKKINNEIRDLLEKLKCSQDCQTNVYFDMIKIYLVDFKKMPYENYDTFIKQYKNSYLSGVDMIRKIEKQIDNP

G382 ... 400 ... 450
VTINAIKFTQKEMGYIIDRFEYHLQKVKHSIDQVTALSDGVKPKQVTKNRLKEYYFNIGNYYSIFKFGKDSLNMLNKALIHKEKIVHNLLGELFGHLEERISKLIDSEYFI

E497 500 ... 550 ... D558 ... Q564
TESNNIISQSEETLKLAEDVYDKNTKLIEDLTLYPHLEINEFKKDYDNNVEDLRESIIYIQSYVSSIKSAYRYNVLEKDSVESKQKNIPANSNAQKKVDELLSIIDSISYS

N591 ... 600
NFSVAENFQKMKDYYKEIEKLKIKILQLIEAIKKYQQHVEELI

b

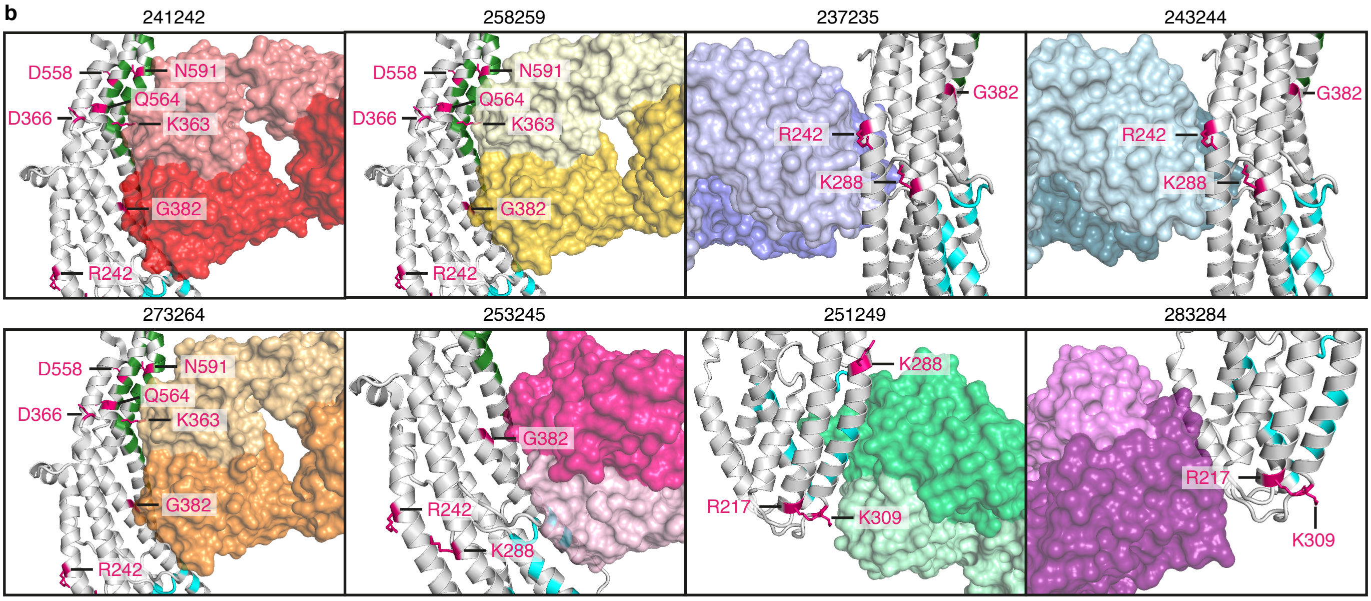

Supplementary Figure S9. PvRBP2b polymorphisms. (a) Sequence of the receptor binding region of PvRBP2b. Polymorphic residues are labelled in pink. **(b)** Mapping of PvRBP2b polymorphisms on X-ray crystal structures. Polymorphisms are colored in pink and labelled. Regions that interact with TfR1 and Tf are highlighted in green and cyan, respectively.

Supplementary Table S1. Primer Table

	Clones	Heavy Gene Forward Primer	Sequence	Heavy Gene Reverse Primer	Sequence
1	**237235**	5' AgeI VH3_33	CTGCAACCGGTGTACATTCTCAGGTGCAGCTGGTGGAG	3' SalI JH 3	TGCGAAGTCGACGCTGAAGAGACGGTGACCATTG
2	**239229**	5' AgeI VH 1/5	CTGCAACCGGTGTACATTCCGAGGTGCAGCTGGTGCAG	3' SalI JH 1/2/4/5	TGCGAAGTCGACGCTGAGGAGACGGTGACCAG
3	**241242**	5' AgeI VH3_23	CTGCAACCGGTGTACATTCTGAGGTGCAGCTGTTGGAG	3' SalI JH 1/2/4/5	TGCGAAGTCGACGCTGAGGAGACGGTGACCAG
4	**243244**	5' AgeI VH3_33	CTGCAACCGGTGTACATTCTCAGGTGCAGCTGGTGGAG	3' SalI JH 3	TGCGAAGTCGACGCTGAAGAGACGGTGACCATTG
5	**250233**	5' AgeI VH3	CTGCAACCGGTGTACATTCTGAGGTGCAGCTGGTGGAG	3' SalI JH 3	TGCGAAGTCGACGCTGAAGAGACGGTGACCATTG
6	**251249**	5' AgeI VH 3_9	CTGCAACCGGTGTACATTCTGAAGTGCAGCTGGTGGAG	3' SalI JH 1/2/4/5	TGCGAAGTCGACGCTGAGGAGACGGTGACCAG
7	**252248**	5' AgeI VH 3_9	CTGCAACCGGTGTACATTCTGAAGTGCAGCTGGTGGAG	3' SalI JH 3	TGCGAAGTCGACGCTGAAGAGACGGTGACCATTG
8	**253245**	5' AgeI VH 1/5	CTGCAACCGGTGTACATTCCGAGGTGCAGCTGGTGCAG	3' SalI JH 1/2/4/5	TGCGAAGTCGACGCTGAGGAGACGGTGACCAG
9	**254255**	5' AgeI VH3_30	CTGCAACCGGTGTACATTCTCAGGTGCAGCTGGTGGAG	3' SalI JH 1/2/4/5	TGCGAAGTCGACGCTGAGGAGACGGTGACCAG
10	**256257**	5' AgeI VH4_34	CTGCAACCGGTGTACATTCCCAGGTGCAGCTACAGCAGTG	3' SalI JH 1/2/4/5	TGCGAAGTCGACGCTGAGGAGACGGTGACCAG
11	**258259**	5' AgeI VH3_23	CTGCAACCGGTGTACATTCTGAGGTGCAGCTGTTGGAG	3' SalI JH 3	TGCGAAGTCGACGCTGAAGAGACGGTGACCATTG
12	**260261**	5' AgeI VH3	CTGCAACCGGTGTACATTCTGAGGTGCAGCTGGTGGAG	3' SalI JH 1/2/4/5	TGCGAAGTCGACGCTGAGGAGACGGTGACCAG
13	**262231**	5' AgeI VH 1/5	CTGCAACCGGTGTACATTCCGAGGTGCAGCTGGTGCAG	3' SalI JH 1/2/4/5	TGCGAAGTCGACGCTGAGGAGACGGTGACCAG
14	**267268**	5' AgeI VH 3_9	CTGCAACCGGTGTACATTCTGAAGTGCAGCTGGTGGAG	3' SalI JH 1/2/4/5	TGCGAAGTCGACGCTGAGGAGACGGTGACCAG
15	**273264**	5' AgeI VH3_23	CTGCAACCGGTGTACATTCTGAGGTGCAGCTGTTGGAG	3' SalI JH 3	TGCGAAGTCGACGCTGAAGAGACGGTGACCATTG
16	**277278**	5' AgeI VH3-53	CTGCAACCGGTGTACATTCCCAGGTGCAGCTGGTGCAG	3' SalI JH 3	TGCGAAGTCGACGCTGAAGAGACGGTGACCATTG
17	**279280**	5' AgeI VH4_34	CTGCAACCGGTGTACATTCCCAGGTGCAGCTACAGCAGTG	3' SalI JH 1/2/4/5	TGCGAAGTCGACGCTGAGGAGACGGTGACCAG
18	**281282**	5' AgeI VH 3_9	CTGCAACCGGTGTACATTCTGAAGTGCAGCTGGTGGAG	3' SalI JH 3	TGCGAAGTCGACGCTGAAGAGACGGTGACCATTG
19	**283284**	5' AgeI VH4_34	CTGCAACCGGTGTACATTCCCAGGTGCAGCTACAGCAGTG	3' SalI JH 1/2/4/5	TGCGAAGTCGACGCTGAGGAGACGGTGACCAG
20	**326327**	5' AgeI VH4_34	CTGCAACCGGTGTACATTCCCAGGTGCAGCTACAGCAGTG	3' SalI JH 1/2/4/5	TGCGAAGTCGACGCTGAGGAGACGGTGACCAG
21	**335338**	5' AgeI VH4-59	CTGCAACCGGTGTACATTCTGAGGTGCAGCTGGTGGAG	3' SalI JH 6	TGCGAAGTCGACGCTGAGGAGACGGTGACCGTG
22	**340341**	5' AgeI VH4-59	CTGCAACCGGTGTACATTCTGAGGTGCAGCTGGTGGAG	3' SalI JH 6	TGCGAAGTCGACGCTGAGGAGACGGTGACCGTG
23	**346343**	5' AgeI VH4-59	CTGCAACCGGTGTACATTCTGAGGTGCAGCTGGTGGAG	3' SalI JH 1/2/4/5	TGCGAAGTCGACGCTGAGGAGACGGTGACCAG

	Clones	Light Gene Forward Primer	Sequence	Light Gene Reverse Primer	Sequence
1	**237235**	5' AgeI V lambda 2	CTGCTACCGGTTCCTGGGCCCAGTCTGCCCTGACTCAG	3' XhoI C lambda	CTCCTCACTCGAGGGYGGGAACAGAGTG
2	**239229**	5' AgeI Vk 3_11	TTGTGCTGCAACCGGTGTACATTCAGAAATTGTGTTGACACAGTC	3' BsiWI Jk 2	GCCACCGTACGTTTGATCTCCAGCTTGGTC
3	**241242**	5' AgeI V lambda 3	CTGCTACCGGTTCTGTGACCTCCTATGAGCTGACWCAG	3' XhoI C lambda	CTCCTCACTCGAGGGYGGGAACAGAGTG
4	**243244**	5' AgeI V lambda 2	CTGCTACCGGTTCCTGGGCCCAGTCTGCCCTGACTCAG	3' XhoI C lambda	CTCCTCACTCGAGGGYGGGAACAGAGTG
5	**250233**	5' AgeI Vk 3_11	TTGTGCTGCAACCGGTGTACATTCAGAAATTGTGTTGACACAGTC	3' BsiWI Jk 1/4	GCCACCGTACGTTTGATYTCCACCTTGGTC
6	**251249**	5' AgeI Vk 3_11	TTGTGCTGCAACCGGTGTACATTCAGAAATTGTGTTGACACAGTC	3' BsiWI Jk 1/4	GCCACCGTACGTTTGATYTCCACCTTGGTC
7	**252248**	5' AgeI Vk 4_1	CTGCAACCGGTGTACATTCGGACATCGTGATGACCCAGTC	3' BsiWI Jk 1/4	GCCACCGTACGTTTGATYTCCACCTTGGTC
8	**253245**	5' AgeI V lambda 2	CTGCTACCGGTTCCTGGGCCCAGTCTGCCCTGACTCAG	3' XhoI C lambda	CTCCTCACTCGAGGGYGGGAACAGAGTG
9	**254255**	5' AgeI Vk 3_15	CTGCAACCGGTGTACATTCAGAAATAGTGATGACGCAGTC	3' BsiWI Jk 3	GCCACCGTACGTTTGATATCCACTTTGGTC
10	**256257**	5' AgeI V lambda 3	CTGCTACCGGTTCTGTGACCTCCTATGAGCTGACWCAG	3' XhoI C lambda	CTCCTCACTCGAGGGYGGGAACAGAGTG
11	**258259**	5' AgeI V lambda 3	CTGCTACCGGTTCTGTGACCTCCTATGAGCTGACWCAG	3' XhoI C lambda	CTCCTCACTCGAGGGYGGGAACAGAGTG
12	**260261**	5' AgeI V lambda 3	CTGCTACCGGTTCTGTGACCTCCTATGAGCTGACWCAG	3' XhoI C lambda	CTCCTCACTCGAGGGYGGGAACAGAGTG
13	**262231**	5' AgeI Vk 3_11	TTGTGCTGCAACCGGTGTACATTCAGAAATTGTGTTGACACAGTC	3' BsiWI Jk 2	GCCACCGTACGTTTGATCTCCAGCTTGGTC
14	**267268**	5' AgeI Vk 1_5	CTGCAACCGGTGTACATTCTGACATCCAGATGACCCAGTC	3' BsiWI Jk 2	GCCACCGTACGTTTGATCTCCAGCTTGGTC
15	**273264**	5' AgeI V lambda 3	CTGCTACCGGTTCTGTGACCTCCTATGAGCTGACWCAG	3' XhoI C lambda	CTCCTCACTCGAGGGYGGGAACAGAGTG
16	**277278**	5' AgeI V lambda 3	CTGCTACCGGTTCTGTGACCTCCTATGAGCTGACWCAG	3' XhoI C lambda	CTCCTCACTCGAGGGYGGGAACAGAGTG
17	**279280**	5' AgeI V lambda 1	CTGCTACCGGTTCCTGGGCCCAGTCTGTGCTGACKCAG	3' XhoI C lambda	CTCCTCACTCGAGGGYGGGAACAGAGTG
18	**281282**	5' AgeI Vk 1_5	CTGCAACCGGTGTACATTCTGACATCCAGATGACCCAGTC	3' BsiWI Jk 1/4	GCCACCGTACGTTTGATYTCCACCTTGGTC
19	**283284**	5' AgeI Vk 1_5	CTGCAACCGGTGTACATTCTGACATCCAGATGACCCAGTC	3' BsiWI Jk 1/4	GCCACCGTACGTTTGATYTCCACCTTGGTC
20	**326327**	5' AgeI Vk 1_5	CTGCAACCGGTGTACATTCTGACATCCAGATGACCCAGTC	3' BsiWI Jk 3	GCCACCGTACGTTTGATATCCACTTTGGTC
21	**335338**	5' AgeI Vk 3_20	TTGTGCTGCAACCGGTGTACATTCAGAAATTGTGTTGACGCAGTCT	3' BsiWI Jk 1/4	GCCACCGTACGTTTGATYTCCACCTTGGTC
22	**340341**	5' AgeI Vk 3_20	TTGTGCTGCAACCGGTGTACATTCAGAAATTGTGTTGACGCAGTCT	3' BsiWI Jk 1/4	GCCACCGTACGTTTGATYTCCACCTTGGTC
23	**346343**	5' AgeI Vk 3_15	CTGCAACCGGTGTACATTCAGAAATAGTGATGACGCAGTC	3' BsiWI Jk 1/4	GCCACCGTACGTTTGATYTCCACCTTGGTC

Supplementary Table S2 | Data collection and refinement statistics for PvRBP2b complexes with PvRBP2b human Fab fragments.

	PvRBP2b-237235 (PDB 6WM9)	PvRBP2b-241242 (PDB 6WN1)	PvRBP2b-243244 (PDB 6WNO)	PvRBP2b-251249 (PDB 6WOZ)	PvRBP2b-253245 (PDB 6WTY)
**Data collection**[a]					
Space group	P 1	C 1 2 1	P 1	P 1 2_1 1	P 1 2_1 1
Cell dimensions					
a, *b*, *c* (Å)	61.90, 86.78, 90.77	360.70, 43.70, 115.33	61.01, 84.44, 90.91	99.57, 163.55, 121.76	70.56, 78.19, 312.08
α, β, γ (°)	91.62, 109.98, 99.88	90.00, 101.69, 90.00	90.73, 110.12, 103.10	90.00, 99.19, 90.00	90.00, 94.16, 90.00
Resolution (Å)	43.67-2.45 (2.51-2.45)	43.72-3.15 (3.23-3.15)	43.00-3.35 (3.44-3.35)	49.14-2.90 (3.07-2.90)	48.70-3.48 (3.69-3.48)
R_{meas}	17.9 (73.6)	17.0 (121.5)	21.6 (98.8)	19.5 (148.4)	85.7 (317.9)[b]
$I/\sigma(I)$	7.4 (1.8)	8.9 (1.3)	6.9 (1.6)	9.8 (1.3)	2.5 (0.5)
$CC_{1/2}$ (%)	98.8 (72.4)	99.4 (57.2)	98.9 (68.5)	99.7 (65.3)	85.0 (13.1)
Completeness (%)	97.4 (96.6)	99.6 (99.9)	97.7 (97.4)	99.4 (96.6)	98.7 (93.1)
Redundancy	3.3 (3.4)	3.8 (3.8)	3.5 (3.5)	7.1 (6.7)	5.4 (5.1)
Wilson *B* (Å^2)	55.2	64.5	68.2	61.0	60.6
**Refinement**					
No. reflections	62,347	31,277	23,187	84,919	43,318
R_{work} / R_{free} (%)	22.3 / 27.9	25.1 / 28.3	24.5 / 28.4	21.1 / 25.8	26.2/30.1
No. atoms					
Protein	11,251	8,910	10,596	22,580	19,922
Water	559	-	-	-	-
B factors					
Protein	33.6	76.5	75.0	61.8	74.7
Water	32.0	-	-	-	-
R.m.s. deviations					
Bond lengths (Å)	0.002	0.002	0.002	0.002	0.001
Bond angles (°)	0.489	0.441	0.456	0.482	0.413
Validation					
MolProbity score	1.36	1.58	1.65	1.45	1.51
Clashscore	4.88	5.84	7.40	4.19	3.60
Poor rotamers (%)	-	-	-	-	-
Ramachandran plot					
Favored (%)	97.5	96.2	96.4	96.3	94.8
Allowed (%)	2.6	3.8	3.7	3.7	5.2
Disallowed (%)	-	-	-	-	-

Supplementary Table S2 | continued

	PvRBP2b-258259 (PDB 6WTV)	PvRBP2b-273264 (PDB 6WTU)	PvRBP2b-283284 (PDB 6WQO)
**Data collection**			
Space group	P 1	P 1	P 2_1 2_1 2_1
Cell dimensions			
a, *b*, *c* (Å)	93.80, 98.65, 103.09	93.99, 99.51, 103.49	88.25, 126.18, 149.63
α, β, γ (°)	114.15, 105.00, 89.69	114.72, 104.98, 89.81	90.00, 90.00, 90.00
Resolution (Å)	45.01-3.05 (3.23-3.05)	49.03-2.55 (2.62-2.55)	42.32-3.15 (3.23-3.15)
R_{meas}	22.6 (65.5)	10.7 (94.3)	27.8 (129.7)
I/σ (I)	5.2 (2.0)	10.0 (1.5)	6.3 (1.4)
$CC_{1/2}$ (%)	97.1 (65.5)	99.7 (64.5)	98.2 (47.4)
Completeness (%)	97.4 (94.9)	98.2 (97.9)	99.3 (97.7)
Redundancy	2.5 (2.5)	3.6 (3.6)	5.5 (5.7)
Wilson *B* (Å^2)	37.8	54.6	52.2
**Refinement**			
No. reflections	60,481	104,773	29,354
R_{work} / R_{free} (%)	23.5 / 27.7	20.8 / 25.5	25.4 / 30.5
No. atoms			
Protein	20,978	22,128	10,482
Water	-	392	-
B factors			
Protein	40.5	55.7	62.3
Water	-	46.7	-
R.m.s. deviations			
Bond lengths (Å)	0.002	0.002	0.002
Bond angles (°)	0.436	0.497	0.467
Validation			
MolProbity score	1.65	1.44	1.68
Clashscore	6.34	4.33	6.62
Poor rotamers (%)	-	-	-
Ramachandran plot			
Favored (%)	95.7	96.5	95.5
Allowed (%)	4.3	3.5	4.5
Disallowed (%)	-	-	-

X-ray diffraction data were collected on single crystals.
[a] Values in parentheses are for highest-resolution shell.
[b] High R_{meas} values are the result of a combination of extending the resolution range to include weak data and the presence of a pseudo-merohedral twin fraction of 0.15.

Supplementary Table S3 | Summary of interactions between PvRBP2b and PvRBP2b human Fab fragments.

PvRBP2b and 237235 Fab fragment based on the crystal structure PDB 6WM9

PvRBP2b	Group	Location	237235 V_H	Group	Distance (Å)
**Hydrogen bonds**					
Asn 174	ND2	N-ter	Asn 106	O	3.0
Arg 193	NH2	β2–α1	Glu 109	O	3.5
Arg 193	N	β2–α1	Glu 109	OE1	3.1
Arg 193	NH2	β2–α1	Asn 110	OD1	3.0
Arg 193	NH1	β2–α1	Asn 110	OD1	3.0
Lys 437	NZ	α7	Tyr 58	OH	3.1
Lys 437	NZ	α7	Ser 61	OG	3.8
Asp 438	OD1	α7	Tyr 58	OH	2.6
Asn 441	ND2	α7	Ser 36	O	3.1
Lys 445	NZ	α7	Ser 36	O	2.8
**Salt bridges**					
Arg 193	NE	β2–α1	Glu 109	OE1	3.8
Arg 193	NE	β2–α1	Glu 109	OE2	4.0
Lys 434	NZ	α7	Asp 62	OD2	2.8
Lys 437	NZ	α7	Asp 59	OD1	3.7
Lys 437	NZ	α7	Asp 59	OD2	3.1

PvRBP2b	Group	Location	237235 V_L	Group	Distance (Å)
**Hydrogen bonds**					
Asn 231	OD1	α2	Thr 101	N	2.9
Asn 231	OD1	α2	Thr 101	OG1	3.8
Asn 231	ND2	α2	Thr 101	OG1	3.5
Arg 235	NH1	α2	Phe 36	O	3.2
Gly 238	O	α2	Asn 38	ND2	2.9
Asn 241	ND2	α2	Asn 38	O	3.2

Other PvRBP2b interfacing residues (237235 V_H)					
Asp 173	Asp 176	Gln 191	Leu 192	His 195	Tyr 200
Asp 203	Arg 207	Lys 244	Lys 248	Lys 334	Asn 444
Ile 448	His 449	Lys 452			

Other PvRBP2b interfacing residues (237235 V_L)					
Thr 171	Asp 173	Arg 193	Pro 194	Ile 199	Tyr 200
Asp 203	Asn 227	Leu 230	Glu 232	Ser 234	Ile 237
Ile 239	Lys 244	Asn 245	Lys 248		

PvRBP2b and 241242 Fab fragment based on the crystal structure PDB 6WN1

PvRBP2b	Group	Location	241242 V_H	Group	Distance (Å)
**Hydrogen bonds**					
Ser 181	O	β1	Arg 35	NH1	3.1
Asp 182	O	β1	Arg 35	NH2	2.3
Lys 278	NZ	α3	Tyr 108	OH	3.1
Glu 279	OE2	α3	Asn 36	ND2	3.8
Tyr 351	OH	α5	Tyr 111	OH	3.0
Lys 379	O	α6	Ser 107	OG	3.6
Gly 382	O	α6	Thr 109	N	3.6
Gly 382	O	α6	Thr 109	OG1	3.4
Asp 386	OD1	α6	Ala 58	N	3.7
Asp 386	OD2	α6	Ser 59	N	3.4
Asp 386	OD1	α6	Ser 59	N	3.4
Asp 386	O	α6	Ser 59	OG	3.3
Asp 386	OD1	α6	Ser 59	OG	2.8
Glu 389	OE1	α6	Ser 57	OG	3.3
Glu 389	OE1	α6	Ala 62	N	3.8

PvRBP2b	Group	Location	241242 V_L	Group	Distance (Å)
**Hydrogen bonds**					
Lys 360	NZ	α5	Lys 35	O	2.5
Lys 363	NZ	α5	Tyr 54	O	2.5
**Salt bridges**					
Asp 356	OD2	α5	Arg 95	NE	3
Lys 360	NZ	α5	Asp 55	OD1	3.5
Lys 360	NZ	α5	Asp 55	OD2	2.6

Other PvRBP2b interfacing residues (241242 V_H)					
Ile 180	Glu 183	Ser 184	Asn 185	Tyr 186	Glu 275
Arg 282	Lys 348	Leu 352	Val 355	Asp 356	Arg 359
Lys 375	Gln 378	Tyr 383	Ile 385	Arg 387	Tyr 390
Gln 393					

Other PvRBP2b interfacing residues (241242 V_L)					
Lys 348	Asn 349	Leu 352	Ser 353	Val 355	Arg 359
Glu 362					

PvRBP2b and 243244 Fab fragment based on the crystal structure PDB 6WNO

PvRBP2b	Group	Location	243244 V_H	Group	Distance (Å)
**Hydrogen bonds**					
Arg 193	N	β2–α1	Glu 109	OE2	2.8
Arg 193	NH2	β2–α1	Asn 110	OD1	2.7
Tyr 200	OH	α1	Asn 62	ND2	3.8
Lys 437	O	α7	Tyr 58	OH	3.2
Asp 438	OD1	α7	Tyr 58	OH	2.4
Asn 441	ND2	α7	Ser 36	O	3.1
Lys 445	NZ	α7	Ser 36	O	3.5
**Salt bridges**					
Arg 193	NH2	β2–α1	Glu 109	OE2	3.9
Lys 437	NZ	α7	Asp 59	OD2	3.7
Lys 437	NZ	α7	Asp 61	OD2	3.5

PvRBP2b	Group	Location	243244 V_L	Group	Distance (Å)
**Hydrogen bonds**					
Asn 231	OD1	α2	Thr 101	N	3.1
Arg 235	NH1	α2	Phe 36	O	3.5
Asn 241	ND2	α2	Asn 38	O	3.1

Other PvRBP2b interfacing residues (243244 V_H)					
Asp 173	Asn 174	Asp 176	Gln 191	Leu 192	HIS 195
Asp 203	Lys 334	Tyr 430			

Other PvRBP2b interfacing residues (243244 V_L)					
Thr 171	Asp 173	Arg 193	Pro 194	HIS 195	Ile 199
Tyr 200	Asp 203	Asn 227	Leu 230	Glu 232	Ser 234
Ile 237	Gly 238	Ile 239	Lys 244	Asn 245	

Supplementary Table S3 | continued

PvRBP2b and 251249 Fab fragment based on the crystal structure PDB 6WOZ

PvRBP2b	Group	Location	251249 V_H	Group	Distance (Å)
Hydrogen bonds					
Tyr 186	OH	β1–β2	Gly 109	N	3.6
Tyr 186	OH	β1–β2	Gly 109	O	2.8
Arg 290	NH1	α3	Tyr 59	OH	3.5
Lys 291	NZ	α3	Tyr 59	O	3.3
Lys 291	NZ	α3	Ser 60	O	3.7
Asp 294	OD2	α3	Thr 57	OG1	2.7
Asp 294	OD2	α3	Gly 58	N	3.4
Asp 294	OD2	α3	Tyr 59	N	2.7
Asp 294	OD2	α3	Ser 60	N	3.7
Asp 294	OD2	α3	Ser 61	N	3.4
Gln 295	NE2	α3	Ser 61	OG	2.8
Gln 295	NE2	α3	Glu 62	OE2	3.7
Lys 297	NZ	α3	Ser 36	O	3.3
Lys 297	NZ	α3	Ser 38	OG	3.8
Asn 300	ND2	α3	Ile 112	O	3.2
Asn 301	OD1	α3	Asp 114	N	2.6
Arg 304	NH1	α3	Asp 114	O	2.8
Asn 428	ND2	α7	Val 110	O	3.7
Salt bridges					
Lys 298	NZ	α3	Glu 62	OE1	3.7
Lys 298	NZ	α3	Glu 62	OE2	2.6

PvRBP2b	Group	Location	251249 V_L	Group	Distance (Å)
Hydrogen bonds					
Glu 308	OE2	α3	Thr 36	OG1	3.1
Salt bridges					
Asp 305	OD1	α3	Arg 96	NH1	3.6
Asp 305	OD1	α3	Arg 96	NH2	2.9
Asp 305	OD2	α3	Arg 96	NH1	3.3
Other PvRBP2b interfacing residues (251249 V_H)					
Asp 182	Glu 183	Ser 184	Tyr 187	Asn 198	PHE 201
Leu 293	Glu 421	PHE 424	Asn 425	Tyr 429	
Other PvRBP2b interfacing residues (251249 V_L)					
Arg 304	Asn 417				

PvRBP2b and 253245 Fab fragment based on the crystal structure PDB 6WTY[a]

PvRBP2b	Group	Location	253245 V_H	Group	Distance (Å)
Hydrogen bonds					
Gln 393	NE2	α6	Ser 112	O	3.1
Salt Bridges					
Asp 386	OD1	α6	Arg 114	NH2	3.5
Glu 389	OE1	α6	Arg 114	NH2	2.5
Glu 389	OE2	α6	Arg 114	NH2	3.1

PvRBP2b	Group	Location	253245 V_L	Group	Distance (Å)
Hydrogen bonds					
None					
Other PvRBP2b interfacing residues (253245 V_H)					
Asp 341	Ile 344	Lys 345	Lys 348	Asn 349	Tyr 351
Leu 352	Val 355	Asp 356	Lys 379	Gly 382	Ile 385
Asp 386	Glu 389	Leu 392	Gln 393		
Other PvRBP2b interfacing residues (253245 V_L)					
Ser 184	Asn 185	Tyr 186	Asp 386	Arg 387	Tyr 390
Gln 393	Lys 394	Lys 396	His 397	Asp 400	Gln 401
Ala 404	Tyr 422	Asn 425	Asn 428	Tyr 429	

PvRBP2b and 258259 Fab fragment based on the crystal structure PDB 6WTV

PvRBP2b	Group	Location	258259 V_H	Group	Distance (Å)
Hydrogen bonds					
Glu 183	O	β1–β2	Ser 80	OG	3.1
Asn 185	N	β1–β2	Ser 80	OG	3.8
Lys 348	NZ	α5	Ser 62	OG	3.2
Lys 348	NZ	α5	Tyr 64	OH	2.3
Tyr 351	OH	α5	Tyr 108	O	2.7
Arg 359	NH1	α5	HIS 112	O	2.5
Asp 386	OD1	α6	Gly 58	N	3.6
Asp 386	OD1	α6	Ser 59	N	3.1
Asp 386	OD2	α6	Ser 59	N	3.2
Asp 386	O	α6	Ser 59	OG	2.5
Asp 386	OD1	α6	Ser 59	OG	2.6
Salt bridges					
Asp 356	OD1	α5	HIS 112	NE2	3.5
Asp 356	OD2	α5	HIS 112	NE2	3.6
Lys 379	NZ	α6	Glu 107	OE2	3.9

PvRBP2b	Group	Location	258259 V_L	Group	Distance (Å)
Hydrogen bonds					
Asp 356	OD1	α5	Thr 36	OG1	3.1
Asp 356	OD2	α5	Thr 36	N	3.7
Other PvRBP2b interfacing residues (258259 V_H)					
Ser 184	Lys 278	Leu 352	Ser 353	Val 355	Gly 382
Tyr 383	Ile 385	Arg 387	Glu 389	Tyr 390	Gln 393
Other PvRBP2b interfacing residues (258259 V_L)					
Lys 348	Asn 349	Leu 352	Arg 359	Lys 360	

Supplementary Table S3 | continued

PvRBP2b and 273264 Fab fragment based on the crystal structure PDB 6WTU

PvRBP2b	Group	Location	273264 V_H	Group	Distance (Å)
Hydrogen bonds					
Tyr 351	OH	α5	Tyr 108	O	2.6
Arg 359	NH2	α5	HIS 112	O	2.5
Asp 386	OD1	α6	Gly 58	N	3.8
Asp 386	OD1	α6	Ser 59	N	3.3
Asp 386	OD2	α6	Ser 59	N	3.5
Asp 386	OD1	α6	Ser 59	OG	3.1
Asp 386	O	α6	Ser 59	OG	3.0
Glu 389	OE1	α6	Ser 62	OG	2.7
Gln 393	NE2	α6	Gly 60	O	3.4
Salt bridges					
Asp 356	OD2	α5	HIS 112	NE2	3.8

PvRBP2b	Group	Location	273264 V_L	Group	Distance (Å)
Hydrogen bonds					
Asp 356	OD2	α5	Thr 35	OG1	3.0
Asp 356	OD1	α5	Ser 36	N	3.6
Asp 356	OD1	α5	Ser 36	OG	2.4
Asp 356	OD2	α5	Ser 36	N	3.4
Lys 360	NZ	α5	Thr 35	O	2.6
Salt bridges					
Lys 360	NZ	α5	Asp 55	OD1	3.5
Lys 360	NZ	α5	Asp 55	OD2	2.5
Other PvRBP2b interfacing residues (273264 V_H)					
Glu 183	Ser 184	Asn 185	Lys 348	Leu 352	Ser 353
Val 355	Lys 379	Gly 382	Tyr 383	Ile 385	Arg 387
Tyr 390					
Other PvRBP2b interfacing residues (273264 V_L)					
Asn 349	Leu 352	Arg 359			

PvRBP2b and 283284 Fab fragment based on the crystal structure PDB 6WQO

PvRBP2b	Group	Location	283284 V_H	Group	Distance (Å)
Hydrogen bonds					
Tyr 211	O	α1	Ser 35	OG	2.3
Tyr 211	OH	α1	Ile 104	O	3.2
Asn 319	ND2	α4	Tyr 37	OH	3.6
Asp 323	OD2	α4	Tyr 37	OH	2.8
Asp 331	OD1	α4	Tyr 108	OH	2.5
Salt bridges					
Lys 216	NZ	α2	Asp 58	OD2	2.4
Asp 323	OD2	α4	Lys 102	NZ	2.8
Asp 323	OD1	α4	Lys 102	NZ	4.0

PvRBP2b	Group	Location	283284 V_L	Group	Distance (Å)
Hydrogen bonds					
Lys 326	NZ	α4	Ser 61	OG	3.8
Lys 333	NZ	α4	Asp 55	O	3.1
Salt bridges					
Lys 326	NZ	α4	Glu 60	OE1	3.7
Lys 326	NZ	α4	Glu 60	OE2	3.5
Lys 333	NZ	α4	Asp 55	OD2	2.5
Other PvRBP2b interfacing residues (283284 V_H)					
Arg 207	Ser 210	HIS 212	Thr 213	Gln 317	Val 320
Met 324	Lys 326	Ile 327	Val 330	Lys 412	
Other PvRBP2b interfacing residues (283284 V_L)					
Val 330	Lys 334	Lys 410			

The distance measurements are based on molecules A, B and C.
Interacting and interfacing residues between PvRBP2b and antibody Fabs was determined using PISA[35].
[a] Some residue side chains are unresolved and the interactions listed here may not be complete.
