# Supplementary material for: Naturally acquired blocking human monoclonal antibodies to Plasmodiumvivax reticulocyte binding protein 2b
Source: Nat Commun. 2021 Mar 9;12:1538. doi: 10.1038/s41467-021-21811-2 (PMC7943553; doi:10.1038/s41467-021-21811-2)
Supplement: Supplementary file 3 — Description of Additional Supplementary Files [file 41467_2021_21811_MOESM3_ESM.pdf]

Supplementary Data 1. Diversity of VDJ gene usage for PvRBP2b human mAbs.

Supplementary Data 2. HDX data table showing for each peptide analyzed and for PvRBP2b alone or in complex with PvRBP2b mouse antibodies 3E9, 6H1 and 10B12: i) deuteration levels expressed as number of deuterons (# D) and ii) percentage deuteration calculated based on a maximal theoretical deuteration level of 85 % (% D). On the right, differences in deuteration levels for between PvRPB2b alone or in presence of any of the mouse antibodies for each peptide are shown. Differences are calculated both in percentage and number of deuterons.

Supplementary Data 3. HDX data table showing for each peptide analyzed and for PvRBP2b alone or in complex with PvRBP2b human mAbs: i) deuteration levels expressed as number of deuterons (# D) and ii) percentage deuteration compared to a highly deuterated sample (% D). Standard-deviation for each of the triplicate measurements are shown.

Supplementary Data 4. Differences in HDX levels for all analysed peptides between PvRBP2b alone and in complex with PvRBP2b human mAbs. Values are expressed as number of deuterons incorporated.

Supplementary Data 5. Differences in HDX levels for all analysed peptides between PvRBP2b alone and in complex with PvRBP2b human mAbs. Values are expressed as percentage peptide deuteration.
