# Supplementary material for: Naturally acquired blocking human monoclonal antibodies to Plasmodiumvivax reticulocyte binding protein 2b
Source: Nat Commun. 2021 Mar 9;12:1538. doi: 10.1038/s41467-021-21811-2 (PMC7943553; doi:10.1038/s41467-021-21811-2)
Supplement: Supplementary file 9 — Reporting Summary [file 41467_2021_21811_MOESM9_ESM.pdf]

nature research

Corresponding author(s): Wai-Hong Tham
Last updated by author(s): Jan 20, 2021

Reporting Summary

Nature Research wishes to improve the reproducibility of the work that we publish. This form provides structure for consistency and transparency in reporting. For further information on Nature Research policies, see our Editorial Policies and the Editorial Policy Checklist.

Statistics

For all statistical analyses, confirm that the following items are present in the figure legend, table legend, main text, or Methods section.

n/a	Confirmed	
☐	☒	The exact sample size (n) for each experimental group/condition, given as a discrete number and unit of measurement
☐	☒	A statement on whether measurements were taken from distinct samples or whether the same sample was measured repeatedly
☐	☒	The statistical test(s) used AND whether they are one- or two-sided<br>*Only common tests should be described solely by name; describe more complex techniques in the Methods section.*
☒	☐	A description of all covariates tested
☐	☒	A description of any assumptions or corrections, such as tests of normality and adjustment for multiple comparisons
☐	☒	A full description of the statistical parameters including central tendency (e.g. means) or other basic estimates (e.g. regression coefficient) AND variation (e.g. standard deviation) or associated estimates of uncertainty (e.g. confidence intervals)
☐	☒	For null hypothesis testing, the test statistic (e.g. F, t, r) with confidence intervals, effect sizes, degrees of freedom and P value noted<br>*Give P values as exact values whenever suitable.*
☒	☐	For Bayesian analysis, information on the choice of priors and Markov chain Monte Carlo settings
☒	☐	For hierarchical and complex designs, identification of the appropriate level for tests and full reporting of outcomes
☒	☐	Estimates of effect sizes (e.g. Cohen's d, Pearson's r), indicating how they were calculated

Our web collection on statistics for biologists contains articles on many of the points above.

Software and code

Policy information about availability of computer code

Data collection

For B cell sorting, cells were sorted on a BD FACS Aria II.
For flow cytometry, data was collected using the BD LSR II flow cytometer.
Antibody affinities were measured using an Octet RED96 instrument.
For FRET data, fluorescence instensity was measured using EnVision plate reader.
For HDX-MS, Mass spectra were acquired on an Orbitrap Velos Pro.
Diffraction data were collected on the MX2 beamline at the Australian Synchrotron.

Data analysis

For flow cytometry, results were analysed using FlowJo software (Three Star; version 10.4.1).
For antibody affinities, curve fitting analysis was performed with Octet Data Analysis software (version 11.1.0.4).
For FRET data, fluorescence measurements were analyzed using Prism software (version 8.4.3).
For HDX-MS, data were analyzed by Mascot (version 2.5) and HD examiner software (Sierra Analytics; version 1.4.3).
Diffraction data were processed with the XDS package (Version Jan 31, 2020 BUILT=20200131) and the probable number of molecules in the asymmetric unit was calculated using an online Matthews probability calculator (http://www.ruppweb.org/mattprob/default.html).
Molecular replacement was performed using Phaser (CCP4 suite; version 7.0.052) and the search for the best starting model was done using the online tool BLASTp (https://blast.ncbi.nlm.nih.gov/).
Structures were manually improved with Coot (version) and refined using Phenix (version 1.14) and interactions between PvRBP2b and antibody Fabs was determined using PISA (CCP4 suite; version 2.1.0).
Structure figures were created using PyMol (version 2.3.0).

For manuscripts utilizing custom algorithms or software that are central to the research but not yet described in published literature, software must be made available to editors and reviewers. We strongly encourage code deposition in a community repository (e.g. GitHub). See the Nature Research guidelines for submitting code & software for further information.

Data

Policy information about availability of data

All manuscripts must include a data availability statement. This statement should provide the following information, where applicable:

- Accession codes, unique identifiers, or web links for publicly available datasets
- A list of figures that have associated raw data
- A description of any restrictions on data availability

P. vivax reticulocyte binding protein 2b constructs were derived from the P. vivax strain Salvador I from the PlasmoDB Database (www.plasmodb.org; accession number: PVX_094255). The structure models used in molecular replacement are from the Protein Data Bank under the following accession codes: 4HWE, 5ODB, 6B0S, 5I16, 6RCO, 4UNU, 6AZM, 5IFH, 6MLK, 5E08, 4LLD and 5NYX. Coordinates and structure factors have been deposited in the Protein Data Bank under accession codes 6WM9 (PvRBP2b-237235 complex), 6WN1 (PvRBP2b-241242 complex), 6WNO (PvRBP2b-243244 complex), 6WOZ (PvRBP2b-251249 complex), 6WTY (PvRBP2b-253245 complex), 6WTV (PvRBP2b-258259 complex), 6WTU (PvRBP2b-273264 complex) and 6WQO (PvRBP2b-283284 complex).

Field-specific reporting

Please select the one below that is the best fit for your research. If you are not sure, read the appropriate sections before making your selection.

- [x] Life sciences
- [ ] Behavioural & social sciences
- [ ] Ecological, evolutionary & environmental sciences

For a reference copy of the document with all sections, see nature.com/documents/nr-reporting-summary-flat.pdf

Life sciences study design

All studies must disclose on these points even when the disclosure is negative.

Sample size	We conducted red blood cell binding assays to determine how antibodies can inhibit binding. From previous publications, we observed that eight biological replicates per condition would provide a reliable indication of whether the antibodies were able to inhibit or not. The FRET assay was performed to determine if the antibodies were able to inhibit complex formation. From our previous publications, we determined that five biological replicates would provide a reliable indication of whether the antibodies were inhibitory or not.
Data exclusions	No data was excluded.
Replication	For the red blood cell binding assay using flow cytometry, we aimed for eight biological replicates with each sample collecting data from 50,000 events. All flow cytometry replications were successful based on the gating controls. For the FRET assay, we aimed for five biological replicates with each replicate performed in triplicate. All FRET replicates were successful based on a FRET signal in the positive control without any addition of antibodies and if the signal was abolished with the addition of denaturing agent (SDS) which served as the negative control.
Randomization	Red blood cells were randomly allocated to each binding condition and the master mix of FRET pairs were aliquoted randomly into each well.
Blinding	Investigators were not blinded during data collection as the red blood cell binding assay was collected by the BD LSR II flow cytometer and the FRET assay readout was performed by an EnVision plate reader.

Reporting for specific materials, systems and methods

We require information from authors about some types of materials, experimental systems and methods used in many studies. Here, indicate whether each material, system or method listed is relevant to your study. If you are not sure if a list item applies to your research, read the appropriate section before selecting a response.

Materials & experimental systems

n/a	Involved in the study	
[ ]	[x]	Antibodies
[ ]	[x]	Eukaryotic cell lines
[x]	[ ]	Palaeontology and archaeology
[x]	[ ]	Animals and other organisms
[ ]	[x]	Human research participants
[x]	[ ]	Clinical data
[x]	[ ]	Dual use research of concern

Methods

n/a	Involved in the study	
[x]	[ ]	ChIP-seq
[ ]	[x]	Flow cytometry
[x]	[ ]	MRI-based neuroimaging

Antibodies

Antibodies used	Anti-PvRBP2b human monoclonal antibodies were recombinantly expressed in the Tham Laboratory at the Walter and Eliza Hall Institute as described in the Materials and Methods. Anti-PvRBP2b mouse monoclonal antibodies, 3E9, 6H1, 10B12, were prepared at the Walter and Eliza Hall Institute Monoclonal Antibody Facility as described previously in Gruszczyk et al., PNAS 2016 and Gruszczyk

	et al., Science 2018.<br>For detection in ELISA, horseradish peroxidase (HRP)-conjugated goat anti-human secondary antibody (Jackson ImmunoResearch; 109-035-088) was used.<br>For detection in flow cytometry, Alexa Fluor 647 chicken anti-rabbit secondary antibody was used (ThermoFisher; A21443).
Validation	Anti-PvRBP2b human monoclonal antibodies are specific to PvRBP2b (Fig.1C) and do not recognize the homologous member in Plasmodium falciparum PfRh4 as shown in Supplementary Fig. 4. The anti-PvRBP2b mouse monoclonals 3E9, 6H1 and 10B12 have been validated and are specific to PvRBP2b as previously described in Gruszczyk et al., Science 2018.<br>Human monoclonal antibody 099100 binds to P. vivax Duffy binding protein (PvDBP) and is characterized in Carias et al., J. Immunol. 2019. Human monoclonal antibody 043038 binds to tetanus toxoid C fragment as described in Carias et al., J. Immunol. 2019.

Eukaryotic cell lines

Policy information about cell lines

Cell line source(s)	Anti-PvRBP2b human monoclonal antibodies were expressed using the Expi293F cell line (ThermoFisher).
Authentication	We did not authenticate this cell line.
Mycoplasma contamination	We did not determine mycoplasma contamination.
Commonly misidentified lines<br>(See ICLAC register)	None were used.

Human research participants

Policy information about studies involving human research participants

Population characteristics	Individual living in Cambodia known to have been previously infected with P. vivax were initially screened for the presence antibodies to P. vivax PvRBP2b. Those individuals who were antibody positive and also have antibodies that blocked binding of PvRBP2b to reticulocytes were used. These were selected from a panel of individuals where we had previously collected PBMC that had been cryopreserved. The cord blood for red blood cell binding assays was obtained through a MTA (ID# M19/110) with the Bone Marrow Donor Institute (BMDI) Cord Blood Bank, as such we are not privy to the population characteristics of their donors.
Recruitment	Participants had been previously recruited for a study on mechanisms naturally acquired immunity to P. vivax in Cambodia. For the binding assays, the donors for cord blood used in the reticulocyte purification are recruited by the BMDI Cord Blood Bank. For our research purposes, we are provided non-bankable cord blood units that would normally be discarded.
Ethics oversight	Institutional review boards from the United States National Institutes of Health (NIAID protocol #08-N094, Clinicaltrials.gov NCT00663546), Cambodian Ministry of Health, and University Hospitals of Cleveland Medical Center approved the protocols (IRB No. 04-14-19) for blood collections. Study protocols were approved by the National Human Research Ethics Committee of the Ministry of Health of Brazil (approval No.551/2010). Written informed consent was obtained from all study participants or their parents/guardians. Human ethics project "14/09, Malaria parasite growth and invasion into reticulocytes" was approved by the Walter and Eliza Hall Institute Human Research Ethics Committee.

Note that full information on the approval of the study protocol must also be provided in the manuscript.

Flow Cytometry

Plots

Confirm that:

- [x] The axis labels state the marker and fluorochrome used (e.g. CD4-FITC).
- [x] The axis scales are clearly visible. Include numbers along axes only for bottom left plot of group (a 'group' is an analysis of identical markers).
- [x] All plots are contour plots with outliers or pseudocolor plots.
- [x] A numerical value for number of cells or percentage (with statistics) is provided.

Methodology

Sample preparation	The biological source of the cells are reticulocytes enriched from whole blood after leukocyte depletion.
Instrument	BD LSR II flow cytometer.
Software	FlowJo software (Three Star; version 10.4.1).
Cell population abundance	Not applicable
Gating strategy	For detection of recombinant protein binding to reticulocytes:<br>Viable cells were gated using foward and side scatter. Negative populations were based on cells incubated with both primary

and secondary antibodies in the absence of recombinant proteins. Negative Thiazole Orange (TO) populations were based on the cells in the absence of Thiazole Orange.

☒ Tick this box to confirm that a figure exemplifying the gating strategy is provided in the Supplementary Information.
